# Monoclonal Antibodies against *Plasmodium falciparum* Circumsporozoite Protein

**DOI:** 10.3390/antib6030011

**Published:** 2017-08-23

**Authors:** Min Zhang, Rajakumar Mandraju, Urvashi Rai, Takayuki Shiratsuchi, Moriya Tsuji

**Affiliations:** 1 HIV and Malaria Vaccine Program, Aaron Diamond AIDS Research Center, Affiliate of The Rockefeller University, New York, NY 10016, USA; zhanmin@iu.edu (M.Z.); mandraju@gmail.com (R.M.); urvashi.rai@gmail.com (U.R.); Shiratsuchi.Takayuki@hq.otsuka.co.jp (T.S.); 2 Department of Pathology, New York University School of Medicine, New York, NY 10016, USA; 3 Department of Pharmacology and Toxicology, Indiana University School of Medicine, Indianapolis, IN 46202, USA; 4 Department of Immunology, UT Southwestern Medical Center Dallas, TX 75390, USA; 5 Otsuka Pharmaceutical Co., Ltd., Osaka 540-0021, Japan

**Keywords:** *Plasmodium falciparum*, circumsporozoite protein, CSP, monoclonal antibody, 2A10, 3C1, 3C2, 3D3

## Abstract

Malaria is a mosquito-borne infectious disease caused by the parasite *Plasmodium* spp. Malaria continues to have a devastating impact on human health. Sporozoites are the infective forms of the parasite inside mosquito salivary glands. Circumsporozoite protein (CSP) is a major and immunodominant protective antigen on the surface of *Plasmodium* sporozoites. Here, we report a generation of specific monoclonal antibodies that recognize the central repeat and C-terminal regions of *P. falciparum* CSP. The monoclonal antibodies 3C1, 3C2, and 3D3—specific for the central repeat region—have higher titers and protective efficacies against challenge with sporozoites compared with 2A10, a gold standard monoclonal antibody that was generated in early 1980s.

## 1. Introduction

In 2015, there were 214 million new cases of malaria (range 149–303 million) and an estimated 438,000 malaria deaths (range 236,000–635,000) worldwide [1]. Malaria is a mosquito-borne disease caused by the protozoan parasite, *Plasmodium* spp. Malaria is transmitted among humans by the bite of female mosquitoes of the genus *Anopheles*. The battle against malaria has been fought using a wide range of interventions, including insecticide-treated bed nets, indoor residual spraying, effective medicines, and vaccine [2,3,4,5]. However, emerging antimalarial drug resistance and insecticide resistance threaten malaria control and public health [6,7,8]. The only approved malaria vaccine is RTS,S/A01 (trade name Mosquirix) to date. RTS,S/A01 represents it’s composed of *P. falciparum* CSP repeat region (R), T-cell epitopes (T) fused to the hepatitis B surface antigen (S) and assembled with un-fused copies of hepatitis B surface antigen, and a chemical adjuvant (AS01) is added to increase the immune system response. The efficacy of RTS,S/AS01 against all episodes of severe malaria is approximately 50% in young children in Africa [9,10,11]. A completely effective vaccine is not yet available for malaria. The novel vectored immunoprophylaxis, an adeno-associated virus-based technology to introduce effective antibody genes in mammalian host, has been added to currently available tools to control malaria [12]. A highly efficient neutralization antibody is one of the essential components of the vectored immunoprophylaxis [12]. Sporozoites are the infectious form of the parasites inside mosquito salivary glands. The circumsporozoite protein (CSP) is a major protein on the surface of *Plasmodium* sporozoites and an immunodominant protective antigen in irradiated sporozoites [13]. The overall structure of CSP is conserved among *Plasmodium* species, consisting of a species-specific central tandem repeat region flanked by conserved N-terminus and C-terminus [14]. The N-terminus is proteolytically processed during sporozoite invasion into host cells, unmasking the C-terminal cell-adhesive domain [15,16]. The C-terminus contains a thrombospondin repeat domain and T cell epitopes. The central repeat region, which is composed of approximately 30 tandem repeats of asn-ala-asn-pro (NANP), corresponds to highly immune-dominant B-cell epitopes [17,18].

The transmission of malaria from mosquito to mammalian host can be prevented by antibodies against CSP, such as the monoclonal antibody (mAb) 2A10 [12,19]. The mouse mAb 2A10 is directed against the central repeat region of *P. falciparum* CSP (PfCSP) [12,20,21,22]. The mouse mAb 2A10 is a useful tool for the study of PfCSP in a mouse model. Delivery of adeno-associated virus expressing 2A10 into mice results in long-lived mAb expression and protection from sporozoite challenge. Vectored immunoprophylaxis provides an exciting new approach to the urgent goal of effective malaria control [12]. However, the mice expressing the CSP-specific mAb 2A10 lower than 1 mg/mL could not be completely protected [12]. Thus, highly potent CSP-specific antibodies are desired for the immunoprophylaxis to control this infectious disease. Here, we report a generation of novel and potent CSP-specific antibodies against PfCSP. In addition, we characterized the mAbs’ subclasses, titers, and protections for sporozoite challenge. Importantly, the protective efficacies of 3C1, 3C2, and 3D3 were found to be better than the reference mAb 2A10.

## 2. Materials and Methods

### 2.1. Expression and Purification of Recombinant PfCSP

PfCSP coding sequence without glycosylphosphatidylinositol (GPI) anchor (GenBank: M19752.1) was amplified using Phusion^®^ high fidelity DNA polymerase (Cat#M0530S, New England Biolabs, Ipswich, MA, USA) with specific primers containing *EcoR* I and *Not* I restriction enzyme recognition sites. The PCR product was purified using Qiagen PCR cleanup kit (Qiagen, Germantown, MD, USA). Both the PCR product and pET20b vector were digested with restriction endonucleases *EcoR* I and *Not* I (New England Biolabs) according to the manufacturer’s protocol. After gel purification, the digested PCR product was ligated into the linearized pET20b vector using Roche rapid DNA ligation kit (Cat. No. 11635379001, Roche, Branford, CT, USA), and then transformed into Top10F’ chemically competent *E. coli.* (Invitrogen, Grand Island, NY, USA) and plated onto Luria-Bertani (LB) agar plates containing ampicillin. A single colony was picked from the plate and inoculated into LB broth plus ampicillin. The recombinant plasmid was purified from the overnight culture using Qiagen plasmid purification kit. The purified plasmid was validated by DNA sequencing and transformed into the BL21(DE3) strain for protein expression. When the culture reached an optical density (OD, 600 nm) of 0.5–0.6, PfCSP expression was induced using IPTG (1 mM) at 20 °C. Then the overnight culture was pelleted by centrifugation and lysed with lysozyme buffer and followed by sonication. Lysate was cleared by centrifugation and the His-tagged PfCSP was purified using Ni^2+^-affinity chromatography (Qiagen, Germantown, MD, USA).

PfCSP purification: 25 mL of nickel nitrilotriacetic acid (Ni-NTA) agarose beads were loaded onto a 22 mL phenyl sepharose column (Pharmacia/Pfizer, New York, NY, USA), washed and equilibrated by 200 mL of His Elution Buffer (50 mM TRIS hydrochloride (Tris-HCl) (pH 8.0), 300 mM imidazole, 50 mM NaCl, 0.1 mM ethylenediaminetetraacetic acid (EDTA), and 1 mM phenylmethane sulfonyl fluoride (PMSF) and 500 mL of His Binding Buffer (50 mM Tris-HCl (pH 8.0), 5 mM imidazole. 100 mM NaCl, 0.1 mM EDTA, and 1 mM PMSF). Then the clarified lysate from 1 L culture was added to the column and washed with 250 mL of His Binding Buffer followed by 500 mL of His Wash Buffer (50 mM Tris-HCl (pH 8.0), 20 mM imidazole. 300 mM NaCl, 0.1 mM EDTA, and 1 mM PMSF). Then, the bound protein was eluted with 20 × 15 mL of His Elution Buffer (50 mM Tris-HCl (pH 8.0), 200 mM imidazole. 300 mM NaCl, 0.1 mM EDTA, and 1 mM PMSF). Proteins were resolved on sodium dodecyl sulfate polyacrylamide gel electrophoresis (SDS-PAGE) followed by Coomassie Brilliant Blue staining. Tris-HCl, EDTA, and PMSF are from Sigma-Aldrich, St. Louis, MO, USA.

### 2.2. Generation of Hybridomas

The recombinant PfCSP was shipped to Green Mountain Antibodies, Inc. (Burlington, VT, USA) for the immunization of mice, followed by the fusion to generate monoclonal antibodies. Briefly, mice were primed with 50 μg of PfCSP emulsified with complete Freund’s adjuvant, followed by weekly immunization of 50 μg of PfCSP emulsified with TiterMax^®^ (Sigma-Aldrich, St. Louis, MO, USA) and SAS^®^ (Sigma-Aldrich) (alternate week). One week after administering seven doses of immunization, the lymph node was isolated. B cells were purified from using anti-B220 magnetic-activated cell sorting (MACS), and then fused with a mouse myeloma cell line. Cloning was achieved by limiting dilution. After re-cloning, positive clones that secrete immunoglobulin G (IgG) against the full-length PfCSP were selected by enzyme-linked immunosorbent assay (ELISA) (Table 1).

### 2.3. ELISA Assay

The ELISA plates were first coated with peptides representing PfCSP N-terminal, the central repeat, or C-terminal regions (10 μg/mL) and then blocked with 3% bovine serum albumin (BSA) in phosphate buffered saline with Tween-20 (PBST) (Table 2). MAbs were 10-fold diluted (0.1–1000 ng/mL) and added to the plates and incubated for 1 h. After washing the plates, horseradish peroxidase (HRP)-conjugated goat anti-human IgG Fc Fragment was added. One hour later, tetramethylbenzidine (TMB) High Sensitivity Substrate was added, and ODs were read at 450 nm. Peptide AIAWAKARARQGLEW was used as a negative control. The mAb 2A10 was used as a positive control [19,23].

### 2.4. Immuno-Fluorescence Assay

1 × 10^4^ salivary gland PfCSP/Py sporozoites were loaded on MP biomedical multi-test glass slides (MP Biomedicals, Santa Ana, CA, USA). PfCSP/Py is an infectious *P. yoelii* parasite bearing a full length of *P. falciparum* circumsporozoite protein (25). After air drying at room temperature, the slides were fixed with 4% paraformaldehyde for 10 min at room temperature, and then blocked with 3% BSA in PBST. The mAbs were two-fold diluted from 1.31 mg/mL to 5 ng/mL, and added to the PfCSP/Py sporozoites-coated wells on the slides for 45 min. After washing with PBS containing 0.05% Tween-20 three times, the slides were incubated with Alexa Fluor 594 conjugate goat anti-mouse IgG (H + L) antibody. One hour later, the slides were washed and mounted in PBS containing 50% glycerol and 1% (*w*/*v*) *p*-phenylenediamine to reduce bleaching.

### 2.5. Sporozoite Neutralization Assays

In vitro neutralization assays were conducted by pre-incubating 2 × 10^4^ PfCSP/Py sporozoites with 100 μg mAb on ice for 45 min, and then adding to 1 × 10^5^ Hepa1-6 cells. Forty-two hours post infection, liver stage parasite burden wear measured by quantitative polymerase chain reaction (qPCR) of *P. yoelii* 18S rRNA as previously described [24]. Mouse glyceraldehyde 3-phosphate dehydrogenase (GAPDH) was used as an internal control. In vivo neutralization assays were conducted by pre-incubating 50 PfCSP/Py sporozoites dissected from infected mosquito salivary glands with 5 or 50 μg mAb on ice for 45 min, and then intravenously injecting into BALB/c mouse. The presence of parasite in blood was determined by Giemsa staining of the blood smear of the recipient mouse.

### 2.6. Giemsa Stain

Starting three days after sporozoite challenge, a drop of blood was collected from the mouse tail vein for thin blood smears on pre-cleaned glass slides. Thin blood smears were fixed with absolute methanol and then stained with diluted Giemsa stain (1:20, *v*/*v*) for 20 min. % parasitemia (% of parasitized red blood cells among total red blood cells) were examined with a 100× oil immersion objective under the microscope.

## 3. Results

### 3.1. Generation of Hybridomas

**Figure 1:**
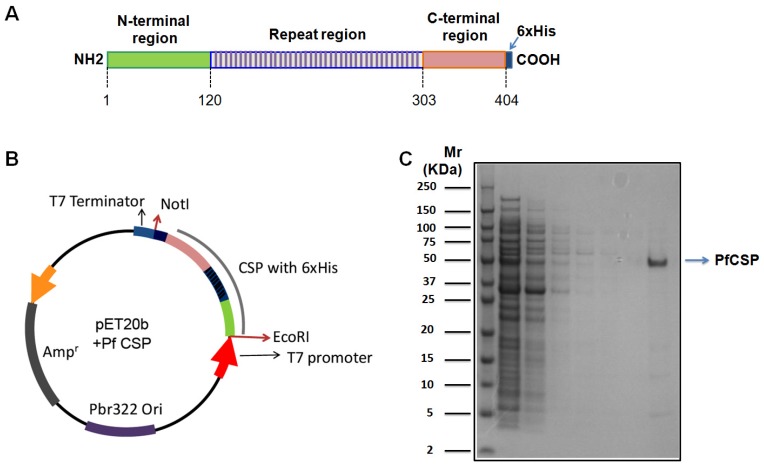
Expression and purification of a recombinant *P. falciparum* circumsporozoite protein (PfCSP). (**A**) Schematic representation of the recombinant PfCSP. Pf CSP coding sequence excluding C-terminal glycosylphosphatidylinositol (GPI) anchor, composed of N-terminal, central repeat, and C-terminal regions, was fused with 6XHis tag at its C-terminus, and cloned into pET20b vector (Stratagene, La Jolla, CA, USA); (**B**) Schematic representation of the PfCSP expression plasmid in this study. The full length of PfCSP without GPI anchor was cloned into pET20b vector between *EcoR* I and *Not* I; (**C**) Expression and purification of a recombinant PfCSP from *E. coli*. The recombinant PfCSP was expressed in BL21 (DE3), and then purified by Ni-Affinity Chromatography. Lane 1, Protein marker; Lane 2, crude extract; Lane 3, flow through; Lane 4–7, washes; Lane 8, elute. Data are representative from three independent experiments.

PfCSP was expressed and purified from *E. coli*. (Figure 1), and then immunized BALB/c mice. The immune spleen cells from the mice producing anti-PfCSP antibodies were fused with myeloma cells, and six hybridoma cell lines (2D4, 3C1, 3C2, 3D3, 4C1, 4C6) were cloned. The mAbs 4C6 2D4 3D3 were identified as belonging to subclass IgG1. The mAbs 3C2 and 4C1 were isotyped as IgG2b class. The mAb 3C1 belonged to subclass IgG3 (Table 1).

### 3.2. Specificity of Anti-PfCSP mAbs

**Figure 2:**
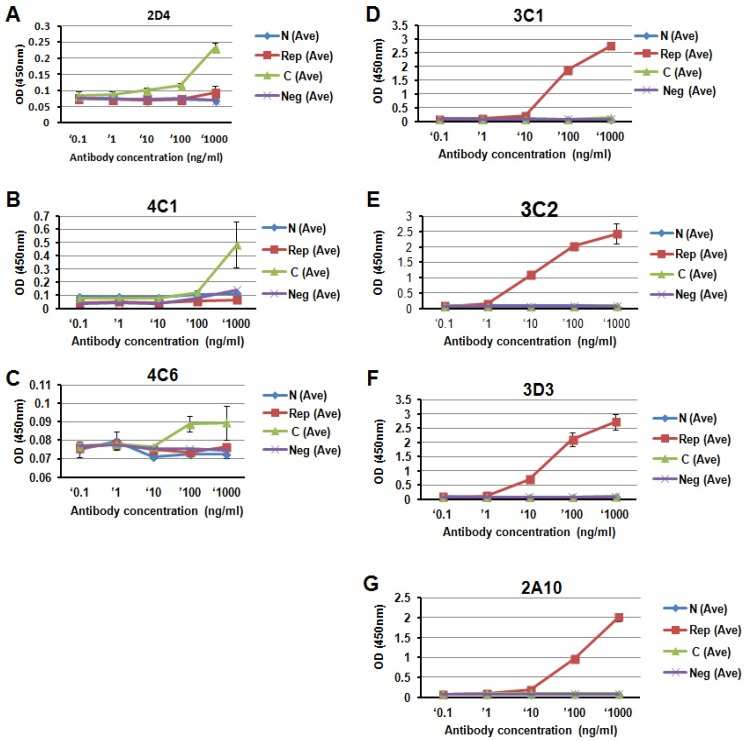
Specificity of anti-PfCSP monoclonal antibodies (mAbs) by enzyme-linked immunosorbent assay (ELISA). Peptides representing PfCSP N-terminal, central repeat, and C-terminal regions were used to evaluate specificity of anti-PfCSP mAbs (Table 1). (**A**), 2D4; (**B**), 4C1; (**C**),4C6; (**D**), 3C1; (**E**), 3C2; (**F**), 3D3; (**G**), 2A10. The mAb 2A10 was used as a positive control. ELISA was performed in duplicate. Data are representative of three independent experiments. OD, Optical density.

The specificity of the mAbs has been explored by measuring their reaction with peptides covering PfCSP N-terminal, the central repeat, and C-terminal regions (Table 2). The mAbs 2D4, 4C1, and 4C6 recognized the PfCSP C-terminal region. The mAbs 3C1, 3C2, and 3D3 recognized the PfCSP central repeat region (Figure 2).

### 3.3. Titration of the PfCSP-Specific mAbs

**Figure 3:**
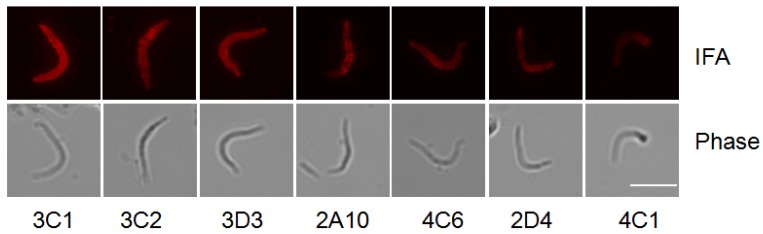
Immunofluorescence assays. PfCSP/Py (a *P. yoelii* parasite bearing *P. falciparum* circumsporozoite protein) salivary gland sporozoites [25] were incubated with 160 ng/mL mAbs, except 4C1 at 328 μg/mL, followed by incubation with Alexa Fluor 594 goat anti-mouse IgG (H + L) *antibody.*

The antibody titer was tested by enzyme-linked immunosorbent assay (ELISA) and immunofluorescence assay (IFA) (Table 1 and Figure 3). ELASA using peptides covering PfCSP showed that the titers of mAbs recognizing the PfCSP central repeat region were higher than those recognizing the PfCSP C-terminal region. The titers of the three mAbs recognizing the PfCSP central repeat were higher than the control 2A10 (3C2 > 3D3 > 3C1 > 2A10). IFA using the *Plasmodium* sporozoites expressing PfCSP [25] also showed that the titers of the mAbs recognizing the PfCSP central repeat were higher than those recognizing the PfCSP C-terminal region. The titer of the three mAbs recognizing the PfCSP central repeat were higher than the control 2A10 (3C1 > 3D3 = 3C2 > 2A10).

### 3.4. Protection of the PfCSP mAbs against PfCSP/Py Sprozoite Challenge

**Figure 4:**
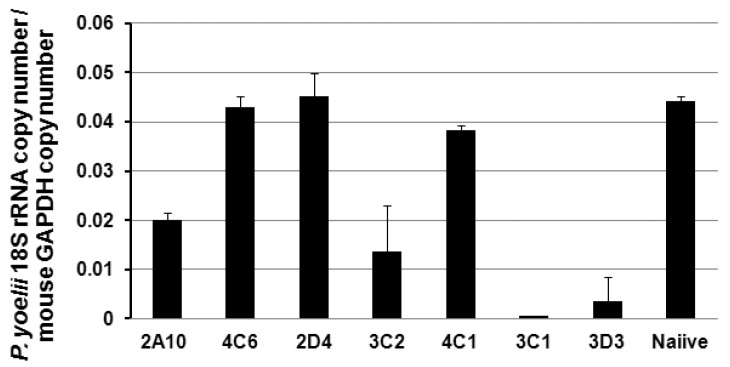
In vitro neutralization assay. 2 × 10^4^ PfCSP/Py sporozoites were pre-incubated with 100 μg of each mAb, and then added to Hepa1-6 cells. Forty-two hours post infection, liver stage parasite burden wear measured by *P. yoelii* 18S rRNA/mouse glyceraldehyde 3-phosphate dehydrogenase (GAPDH). Naive mouse serum was used as control. The in vitro neutralization assay was performed in triplicate. Data are representative of two independent experiments.

**Figure 5:**
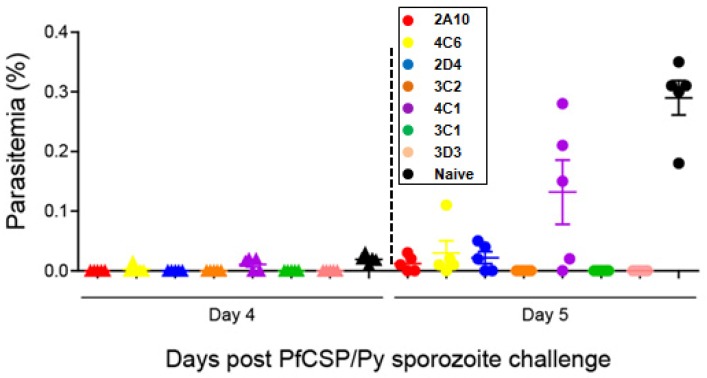
Parasitemia of mice in the neutralization assay. Fifty PfCSP/Py sporozoites were incubated with 50 μg mAbs followed by i.v. injection into BALB/c mice (five mice per group). Parasitemia were counted by Giemsa stains of mouse tail blood followed by microscopy. Data are parasitemia of the mice four and five days post challenge. Naive mice were i.v. injected with 50 PfCSP/Py sporozoites as positive controls.

We then examined the protection of the PfCSP mAbs against malaria sporozoite challenge in vitro and in vivo. For the malaria sporozoite challenges, we used the highly infectious hybrid PfCSP/Py sporozoite, which is based on rodent *P. yoelii* parasite and its CSP is replaced by the full-length of CSP from *P. falciparum* [25]. We found that mAb 3C1, 3C2, and 3D3 significantly inhibited the parasite development in Hepa 1–6 cells compared with 2A10, which is an effective mouse mAb specific for the PfCSP central repeat [19,23] (Figure 4). This was in agreement with the in vivo neutralization assay (Table 3 and Figure 5). Fifty μg of 3C1, 3C2, and 3D3 completely protected the mice from PfCSP/Py sporozoite challenge. The protective effect of 3C1, 3C2, and 3D3 were better than the previously generated mAb 2A10. Even 5 μg of 3C1 partially protected the challenged mice compared to the mAb 2A10.

## 4. Discussion

The CSP consists of the N-terminal flanking region, the central region that contains repetitive immunodominant B-cell epitopes, and the C-terminal flanking region that contains multiple T-cell epitopes. The N-terminus of the CSP is proteolytically processed during the sporozoite invasion into host cells [15,16]. This may explain why we did not obtain specific antibodies against the N-terminus. The abundant NANP repeats present within the central region are likely to contribute to the high neutralization efficacies of the mAbs against PfCSP central repeat region, as previously published [26,27]. In fact, mAbs, which recognize the PfCSP central repeat region, have been shown to exert a potent neutralization activity against the sporozoites [26,27]. All the mAbs 3C1, 3C2, 3D3, and 2A10, recognize the central repeat region of the PfCSP. Fifty μg of the novel mAbs 3C1, 3C2, and 3D3 completely protected the mice from PfCSP/Py sporozoites challenge; while the reference mAb 2A10 only partially protected the mice. A likely explanation is that the native structure of the central repeats of the CSP is not in a random coil state, and the repeat region is predicted to form a rod-like structure [28]. It is speculated that these mAbs recognize structurally different epitopes coded by NANP repeat, resulting in different protection efficacies. The titers (3C1 > 3D3 = 3C2 > 2A10) of these novel mAbs determined by IFA using a whole malaria parasite (sporozoite), as an antigen, corroborate their protection efficacies in vitro (3C1 > 3D3 > 3C2 > 2A10), as well as in mice (3C1 > 3D3 = 3C2 > 2A10). These indicate that mAbs having higher titers against the native from of the CSP expressed by sporozoites exert higher protection efficacies.

Synthesized peptides and sporozoites were used in ELISA and IFA, respectively, to determine the antibody titers. Sporozoites express a native form of the PfCSP, whereas synthesized peptides represent the primary structure of PfCSP. B-cell epitopes are typically classified as either linear epitopes or conformational epitopes, which constitute the spatially folded amino acids and lie far away in the primary sequence. The difference seen by ELISA and IFA may reflect the structural properties of unique B-cell epitopes recognized by our mAbs.

Over the past few years, there has been growing interest in use of vectored immunoprophylaxis to protect hosts from HIV. Vectored immunoprophylaxis is based on adeno-associated virus (AAV) as a vehicle for generating the existing anti-HIV neutralizing antibodies in humans [29,30]. Recently vectored immunoprophylaxis has been utilized for other diseases including malaria and colorectal cancer [12,31]. This new tool requires potent neutralizing antibodies. Although human monoclonal antibodies against PfCSP have been generated, only one mouse mAb against PfCSP, 2A10, has been used as a gold standard mAb for more than three decades. It is noteworthy that a few new mouse mAbs against PfCSP, which we generated in this study, are found to be more potent than 2A10. Therefore, we believe it is important to assess the characteristics of these newly generated mAbs before humanizing them for the purpose of clinical applications, such as a vectored immunoprophylaxis, in the future. Moreover, the mouse mAbs generated in this study are useful tools for the study of PfCSP in a mouse model.

## 5. Conclusions

In summary, here we report a generation of novel mAbs specific against the CSP from *P. falciparum.* The mAbs 2D4, 4C1, and 4C6 recognize the C-terminal region of PfCSP. The mAbs 3C1, 3C2, and 3D3 recognize the central repeat region of PfCSP, and their titers and protection efficacies are higher than 2A10, which has been widely used as a gold standard antibody against PfCSP.

## Figures and Tables

**Table 1:** The titers of the PfCSP-specific mAbs *.

Name of the mAb	Titer (IFA)	Titer (ELISA)	Subclass
2A10	40 ng/mL	10 ng/mL	IgG2a
4C6	80 ng/mL	1 μg/mL	IgG1
2D4	80 ng/mL	500 ng/mL	IgG1
3C2	10 ng/mL	1 ng/mL	IgG2b
4C1	328 μg/mL	200 ng/mL	IgG2b
3C1	5 ng/mL	5 ng/mL	IgG3
3D3	10 ng/mL	2 ng/mL	IgG1

* PfCSP: *P. falciparum* circumsporozoite protein; IgG: immunoglobulin G; IFA: immunofluorescence assay; mAb: monoclonal antibody.

**Table 2:** Synthetic peptides representing PfCSP.

Peptide ID #	Sequence	Position
1	MMRKLAILSVSSFLF	N-terminus
2	SSFLFVEALFQEYQC	N-terminus
3	QEYQCYGSSSNTRVL	N-terminus
4	NTRVLNELNYDNAGT	N-terminus
5	DNAGTNLYNELEMNY	N-terminus
6	LEMNYYGKQENWYSL	N-terminus
7	NWYSLKKNSRSLGEN	N-terminus
8	SLGENDDGNNEDNEK	N-terminus
9	EDNEKLRKPKHKKLK	N-terminus
10	HKKLKQPADGNPDP	N-terminus
11	NANPNVDPNANPNVD	Repeats
12	NPNVDPNANPNVDPN	Repeats
13	NVDPNANPNANPNAN	Repeats
14	NPNANPNANPNANPN	Repeats
15	NANPNANPNANPNAN	Repeats
16	NANPNANPNANPNVD	Repeats
17	NPNVDPNANPNANPN	Repeats
18	NANPNANPNKNNQGN	Repeats
19	NNQGNGQGHNMPNDP	C-terminus
20	MPNDPNRNVDENANA	C-terminus
21	ENANANSAVKNNNNE	C-terminus
22	NNNNEEPSDKHIKEY	C-terminus
23	HIKEYLNKIQNSLST	C-terminus
24	NSLSTEWSPCSVTCG	C-terminus
25	SVTCGNGIQVRIKPG	C-terminus
26	RIKPGSANKPKDELD	C-terminus
27	KDELDYANDIEKKIC	C-terminus
28	EKKICKMEKCSSVFN	C-terminus
29	SSVFNVVNSSIGLIM	C-terminus
30	IGLIMVLSFLFLN	C-terminus
31	AIAWAKARARQGLEW	Negative Control Peptide

**Table 3:** In vivo neutralization assay.

Amount of mAb	5 μg				50 μg			
Days Post Challenge	Day 3	Day 4	Day 5	Day 14	Day 3	Day 4	Day 5	Day 14
2A10	0 *^,#^	4	5	5	0	0	3	3
4C6	0	5	5	5	0	1	4	4
2D4	0	4	5	5	0	0	3	3
3C2	0	1	5	5	0	0	0	0
4C1	0	5	5	5	0	3	4	4
3C1	0	1	4	4	0	0	0	0
3D3	0	2	5	5	0	0	0	0
Naiive	0	5	5	5	0	5	5	5

* Five mice per group. ^#^ The number of infected mice.
